# Monitoring Winter Stress Vulnerability of High-Latitude Understory Vegetation Using Intraspecific Trait Variability and Remote Sensing Approaches

**DOI:** 10.3390/s20072102

**Published:** 2020-04-08

**Authors:** Elmar Ritz, Jarle W. Bjerke, Hans Tømmervik

**Affiliations:** 1 Institute for Water and River Basin Management, Department of Aquatic Environmental Engineering, Karlsruhe Institute of Technology, Gotthard-Franz-Str. 3, 76131 Karlsruhe, Germany; elmar.ritz@kit.edu; 2 Norwegian Institute for Nature Research, FRAM–High North Research Centre for Climate and the Environment, P.O. Box 6606 Langnes, NO-9296 Tromsø, Norway; jarle.bjerke@nina.no

**Keywords:** climate change, evergreen plants, extreme events, flavonol and chlorophyll sensor (Dualex), greenness indices, mosses, near-remote sensing active and passive NDVI sensors, Sentinel-2, subarctic vegetation damage

## Abstract

In this study, we focused on three species that have proven to be vulnerable to winter stress: *Empetrum nigrum,* *Vaccinium vitis-idaea* and *Hylocomium splendens.* Our objective was to determine plant traits suitable for monitoring plant stress as well as trait shifts during spring. To this end, we used a combination of active and passive handheld normalized difference vegetation index (NDVI) sensors, RGB indices derived from ordinary cameras, an optical chlorophyll and flavonol sensor (Dualex), and common plant traits that are sensitive to winter stress, i.e. height, specific leaf area (SLA). Our results indicate that NDVI is a good predictor for plant stress, as it correlates well with height (*r* = 0.70, *p* < 0.001) and chlorophyll content (*r* = 0.63, *p* < 0.001). NDVI is also related to soil depth (*r* = 0.45, *p* < 0.001) as well as to plant stress levels based on observations in the field (*r* = −0.60, *p* < 0.001). Flavonol content and SLA remained relatively stable during spring. Our results confirm a multi-method approach using NDVI data from the Sentinel-2 satellite and active near-remote sensing devices to determine the contribution of understory vegetation to the total ecosystem greenness. We identified low soil depth to be the major stressor for understory vegetation in the studied plots. The RGB indices were good proxies to detect plant stress (e.g. Channel G%: *r* = −0.77, *p* < 0.001) and showed high correlation with NDVI (*r* = 0.75, *p* < 0.001). Ordinary cameras and modified cameras with the infrared filter removed were found to perform equally well.

## 1. Introduction

Global warming will affect arctic and subarctic regions more than any other area in the world [1]. It is expected to increase the productivity of subarctic and arctic ecosystems [2,3,4]. Increasing productivity and biomass is generally known as ‘greening’ [5]. Major drivers are a longer growing season and increasing summer warming [6]. However, negative trends in productivity and biomass, known as ‘browning’, have also been reported [6]. For the Arctic as a whole, trends are complex, as Myers-Smith et al. state: “Figures vary from 42% greening and 2.5% browning from 1982 to 2014 in the GIMMS3g AVHRR dataset to 20% greening and 4% browning from 2000 to 2016 in Landsat data, and to estimates of 13% greening and 1% browning for the MODIS trends calculated for 1,000 random points in the tundra polygon from 2000 to 2018.” ([7], p. 107).

In the subarctic region of Scandinavia, i.e. Norway, Finland, and Sweden north of the Arctic Circle, the main drivers of browning are winter warming events and pest outbreaks [8]. Winter warming events can melt the insulating snow cover that normally protects photosynthetic short-statured organisms overwintering with aboveground tissue (e.g. prostrate shrubs, cushion plants, bryophytes, and lichens) from the harsh ambient winter weather conditions. After a few thaw days, ground vegetation becomes exposed to ambient air and hibernation is interrupted, thus reducing the protection of photosynthetic organisms against frost, which may easily lead to freezing damage upon return of normal winter weather [9]. Soil communities, including both micro-arthropods and bacteria, can also be severely affected [10]. Overall, a warmer winter climate changes species compositions and reduces carbon cycling [11]. In subarctic and arctic regions evergreen plants in particular are sensitive to changing winter climate and reduced snow cover [12]. This includes the widespread dwarf shrubs *Empetrum nigrum* L., *Vaccinium vitis-idaea* L., *Cassiope tetragona* (L.) D.Don, and *Calluna vulgaris* (L.) Hull, as well as the tall coniferous shrub *Juniperus communis* L. [6,11,13]. Bryophytes, such as the widespread feathermoss *Hylocomium splendens* (Hedw.) Schimp., deciduous shrubs, such as *Vaccinium myrtillus* L., evergreen horsetails (*Equisetum* spp.), as well as small cushion plants show reduced growth following exposure to winter warming [9,11,13,14]. The other major factor causing browning are pest outbreaks. Recently, increasing frequency and intensity of outbreaks of leaf-defoliating geometrid moths led to massive canopy defoliation of their preferred host tree *Betula pubescens* Ehrh. and understory plants [13,15,16]. Overall, multiple stress events are main drivers of browning. Given the high focus on climate change-induced changes in northern primary productivity, it is important to develop easy and reliable methods for assessment of plant vitality.

For a long time, satellites have monitored the global vegetation status [2,3,17]. Spectral sensors operated near the target vegetation are increasingly applied for assessing the plant status [18,19]. However, near-remote time series of the plant status are still uncommon, which is partly due to the need for expensive equipment, for example spectroradiometers [20]. In recent years, several new and low-cost active and passive proximal sensors were developed. This includes sensors measuring the normalized difference vegetation index (NDVI). NDVI is a radiometric measure of the amount of radiation (≈∼400–700 nm) absorbed by vegetation during photosynthesis. It is calculated from contrasting reflectance at near-infrared (NIR) and red bands [21,22].

NDVI has been widely used in studies of phenology, productivity, biomass, and disturbance monitoring, as it has proven to be a good proxy of the vegetation’s photosynthetic activity [19,23]. NDVI works well for subarctic ecosystem monitoring and is widely used on different scales and as a vegetation marker [24,25,26].

Previously, modified cameras—with the infrared filter removed—were found to be good NDVI surrogates. In such cameras, the NDVI proxy is commonly calculated by using the enhanced red channel and the blue channel (BNDVI) [27]. However, a combination of the enhanced red channel and the green channel might also be of interest due to a strong linear correlation with the chlorophyll content (GNDVI) [28]. Additionally, ordinary cameras were increasingly applied for vegetation analysis and phenology studies in recent years. Greenness indices based on ordinary RGB images from such cameras are promising NDVI substitutes [28,29], even for high-arctic vegetation [30].

In subarctic forests, the contribution of understory vegetation (i.e. dwarf shrubs, herbs, graminoids, bryophytes and lichens) to the total ecosystem productivity is similar to that of trees [31]. Moreover, biodiversity of vascular plants at high latitudes is relatively low, which makes research into dominant species and their vulnerability to environmental change even more important [32]. We hypothesized that in situ estimates of plant damage would be correlated to optical measurements of plant greenness, but that greenness indices would vary in their explanatory power. Our second hypothesis was that plant stress would vary over short distances in a rolling subarctic landscape and that this would be detectable both by near-remote sensing measurements and by Sentinel. To this end, we combined near-remote sensing approaches with classical determination of plant traits of understory vegetation to address the following research questions:

(1) What is the range of intraspecific variability of common traits of dwarf shrubs and mosses in subarctic spring?
(2) Which traits are reliable indicators of plant stress?
(3) How do the indices derived from ordinary and modified RGB cameras correlate with common plant traits?

To answer these questions, we made analyses in a widespread subarctic heath ecosystem, focusing on vegetation plots dominated by two evergreen dwarf shrubs and a mat-forming moss.

## 2. Materials and Methods

### 2.1. Site Description

We selected plots in wind-exposed areas where snow cover generally is shallow and plants are more susceptible to winter frost-thaw stress. Eighteen plots (1 m × 1 m) were assessed within a total area of approx. 1 km^2^ in Tromsø, Troms County, northern Norway (Figure S1, Table S1, Figure S2b,c). Satellite upscaling was conducted in homogeneous plots within a wider area in Tromsø (Table S2), from 8–12 June 2017, corresponding to days of year (DOY) 159–163. The three areas (a total of ca. 0.5 ha) for the upscaling approach were not congruent to the eighteen field plots (a total of 18 m^2^). We chose three separate areas in order to avoid trails, snow patches, unvegetated ground and rocky steep slopes (as exemplified in Figure S2a). Unvegetated ground was estimated to be around 10% in the area shown in Figure S2a. The other two areas had around 5% of unvegetated ground. Satellite upscaling was performed when snow patches became sparse, but before budburst of the deciduous trees in the heath. The study focuses on the evergreen dwarf shrub species *Empetrum nigrum* and *Vaccinium vitis-idaea* and the mat-forming moss *Hylocomium splendens*. These species are abundant, co-occur in boreal ecosystems, and are linked to browning [9,14,33]. Our continuous monitoring of plant vitality in the study area shows that these species have not been exposed to severely stressful events since 2012, as reported in Bjerke et al. [15]. Minor damage rates were recorded in more recent years, then mostly restricted to wind-exposed sites with little snow accumulation (unpublished observations). Thus, plant traits in the study area were expected to vary naturally along microclimatic gradients. Plots with different stress levels (or health states) were established for each species to monitor natural intraspecific trait variabilities. The studied plots were dominated by (with number of plots in parentheses): *V. vitis-idaea* (two), *H. splendens* (three), *E. nigrum* (nine), and mixed plots of *E. nigrum* with a lower layer of *H. splendens* (four).

### 2.2. Data Collection

Five greenness measurements were made in each plot (*n* = 87) from DOY 130 to DOY 180. Weather conditions varied between days of measurements. For greenness measurements we used four handheld spectral devices (Table 1). The Mapir NDVI camera was only accessible in the last two sampling cycles (*n* = 35). All passive spectral devices were applied 1.5 to 2.0 m above the plot for photographing. We avoided photographing direct light reflectance in the calibration target, and also avoided overexposure. The active Greenseeker sensor measurements were acquired 60 cm above ground.

Measurements of epidermal chlorophyll and flavonol content were conducted with the optical Dualex 4 scientific instrument (Force-A, Orsay, France). The readings of this instrument show a linear relationship to chlorophyll concentrations calculated from extractions. Readings are given in μg cm^−2^, and the measuring wavelength for chlorophyll is a ratio of transmittance at 710 and 850 nm [34]. Within each plot, we sampled at five different spots. The sampling dates were the same as for the greenness measurements. Measurements were made on the newest, fully developed segment of *H. splendens* and on shoot tips of *E. nigrum*. Eight *V. vitis-idaea* leaves of one plant were measured per sample, starting with the upper (= newer) leaves. Hence, we measured physiological traits in different health states on three plots per species (nine in total). All measurements were performed with the adaxial setting of the device. High correlations with the abaxial side are found [35]. Measurements of *V. vitis-idaea* leaves showed reliable results. However, for the shoots of *H. splendens*, stable and reproducible results could only be achieved when three shoots were stacked and fixed with a transparent tape (Figure S3b). The same process was used for the shoots of *E. nigrum* (Figure S3a). At least eight chlorophyll measurements were performed on each sample and readings were then averaged and divided by the number of stacks in the tape. This results in 40 measurements per sampling date and plot. Palta [36] identified leaf anatomy, leaf veins, the presence of other pigments, and leaf thickness as main causes of large variations in chlorophyll meter readings. Hence, we decided to take the specific leaf area (SLA) into account. SLA is also of additional value to determine growth and plant stress [37,38]. After the chlorophyll measurements had been performed, a 6 mm circle was punched out of the prepared samples (Figure S3) [39]. First, fresh weight of the samples was measured. Then, the samples were dried for 24 h at 70 °C before dry weight was measured. This provided information on moisture content and SLA. Plant height was measured as median height above ground. Plant height of *H. splendens* refers to the thickness of the moss layer. We assessed stress levels for each plot in the first and last sampling periods. The stress estimate is a bare-eye classification of visibly dead or dying leaves versus healthy leaves of evergreens within small plots. Leaves that are dead or dying are brown, while healthy leaves are green. The stress estimate thus ranges from 0 to 100%, and it has turned out to be closely correlated to NDVI [13] and CO_2_ fluxes [40].

### 2.3. Data Processing

#### 2.3.1. Greenness Indices

We applied four different devices and extracted six different greenness indices from these (Table 1). The calibration methods applied are also listed. The Greenseeker did not need a calibration; internal tests suggest that measurements are not dependent on environmental changes [41].

**Table 1:** Background information on the greenness indices used in this study. Market prices refer to price levels in 2019.

Name	Equation	Device	Market Price	Calibration	Comments	Source
Greenseeker NDVI	Automatically calculated NDVI output; range 0–1 ^1^	GreenSeeker handheld crop sensor (Trimble Inc., Sunnyvale, CA, USA)	ca. 700 $	Not needed	Active sensor	[41,42]
Mapir NDVI	$\frac{\mathrm{NIR}-\mathrm{Red}}{\mathrm{NIR}+\mathrm{Red}}\;$	Survey2 Camera – NDVI (Mapir Inc., San Diego, CA, USA)	ca. 400 $	Mapir target	Passive sensor	[43]
BNDVI	$\frac{\mathrm{Red}-\mathrm{Blue}}{\mathrm{Red}+\mathrm{Blue}}\;$	Modified Canon Eos 450D (no IR filter) (MaxMax, LDP LLC, Carlstadt, CA, USA)	Body + conversion = 250 $	White balance (WB) on gray area ^2^	Widely used NDVI surrogate	[44]
GNDVI	$\frac{\mathrm{Red}-\mathrm{Green}}{\mathrm{Red}+\mathrm{Green}}\;$	Same as for BNDVI	Same as for BNDVI	White balance (WB) on gray area ^2^	More linear correlation with chlorophyll than NDVI	[28,29]
GRVI	$\frac{\mathrm{Green}-\mathrm{Red}}{\mathrm{Green}+\mathrm{Red}}$	α7 (ILCE-7) (Sony Corp., Tokyo, Japan)	ca. 700 $, but could be any RGB camera	3-step gray card ^2^, when EV = −0.7	RGB index	[45]
Channel G%	$\frac{\mathrm{Green}}{\mathrm{Red}+\mathrm{Green}+\mathrm{Blue}}$	Same as for GRVI	Same as for GRVI	3-step gray card and white balance on white area ^2^ when EV = −0.7	RGB index	[46,47]

^1^ According to the manufacturer’s documents, even non-chlorophyll-containing surfaces, such as soil, have small NDVI values. Therefore, values below 0.15 are rarely measured. Likewise, normal NDVI, with a range from −1 to 1, shows NDVI values between 0.00 and 0.12 for scarce vegetation or bare soil. ^2^ Ordinary gray card with (N2, N5, N9.5) with an accuracy of about 5% on the Macbeth color space.

The spectral properties of the devices we used for NDVI calculation are listed in Table 2. The active Greenseeker device has very similar bandwidths and -peaks to the Sentinel-2 bands 4 and 7. The spectral properties of the passive Mapir camera are closer to the “normal” NDVI calculated with Sentinel-2 bands 4 and 8.

#### 2.3.2. Analysis of the Data

For the calibration of the RGB indices as well as of BNDVI and GNDVI, an ordinary gray card was applied. The card is printed on Teslin Synthetic (greywhitebalancecolorcard, Northfleet, UK]. According to the manufacturer, it has an accuracy of 5% on the Macbeth color space. To decide whether a three-step reflectance calibration on black, gray, and white is superior to a normal white balance, the Channel G% index was calculated for both calibrations. For the Mapir camera (Mapir Inc., San Diego, CA, USA) conversion and calibration were performed with the Mapir calibration target V1 and the QGIS software plug-in version 1.1.2. (Mapir Inc.). All other calibrations and NDVI calculations were performed in WINCAM pro 2013a (Regent Instruments Inc., Quebec City, QC, Canada). Further processing was done in EXCEL (Microsoft Corp., Redmond, WA, USA), while statistical analyses were performed with SPSS 25.0. (IBM Corp., Armonk, NY, USA). Additional, logistic curve fitting was analyzed using the Excel add-on Xlfit version 5.3.1.3 (ID Business Solutions Ltd., Guildford, UK). We calculated all correlations with a two-tailed Pearson´s testimony. Percentiles are weighted averages. Satellite NDVI data were retrieved from ESA’s Sentinel-2 Open Access Hub [https://scihub.copernicus.eu]. Sentinel-2 satellite images were analyzed using ESA’s SNAP software version 5.0.8 with the integrated Sentinel-2 Toolbox (ESA, Common Service Section, Rome, Italy). Atmospherically corrected 2A products from DOY 159 and DOY 163 in 2017 were used. Cloud cover was below 1% and products were analyzed with a resampled spatial resolution of 20 m. Downscaling was done with the “mean method,” which calculates the output as mean of every source pixel value. The downscaling was performed to compare the different bands of Sentinel-2 for a pixelwise NDVI comparison, and also to reduce small-scale effects, like imprecise GPS coordinates (up to 3 m). To compare spaceborne and handheld NDVI data, at least four GPS waypoints were taken per area and NDVI values between the waypoints were measured with the Greenseeker handheld sensor (Trimble Inc., Sunnyvale, CA, USA, see Table S2). The sensor can also be used for measurement over a larger area. Then, it calculates an average of the scanned area. The Sentinel-2 image pixels corresponding to the GPS waypoints were identified and values were compared with the Greenseeker data.

## 3. Results

### 3.1. Descriptive Statistics

Descriptive statistics of the vegetation indices and plant traits are listed in Table 3. Due to relatively large sampling sizes (*n > 36*) for all traits on plot level we can assume that data is normally distributed. The combination of three different species in one dataset (Table 3), might lead to less normal distributed data. According a Kolmogorov-Smirnov test, all datasets of Table 1 are normally distributed (*p* > 0.05), except for Channel G%, Plant height, Stress level, Soil depth, SLA and Flav. However, a species-specific normal distribution is achieved for Plant height, NBI, SLA and Flav. For Channel G% the species-specific normal distribution does not hold for *V. vitis-idaea*, which was monitored on only two plots with highly contrasting stress levels. Stress level in total is not expected to be normally distributed due to plot selection by contrasting health states. Moreover, Soil depth is also not expected to be normally distributed.

### 3.2. Comparison of Different Vegetation Indices and Plant Traits

**Figure 1:**
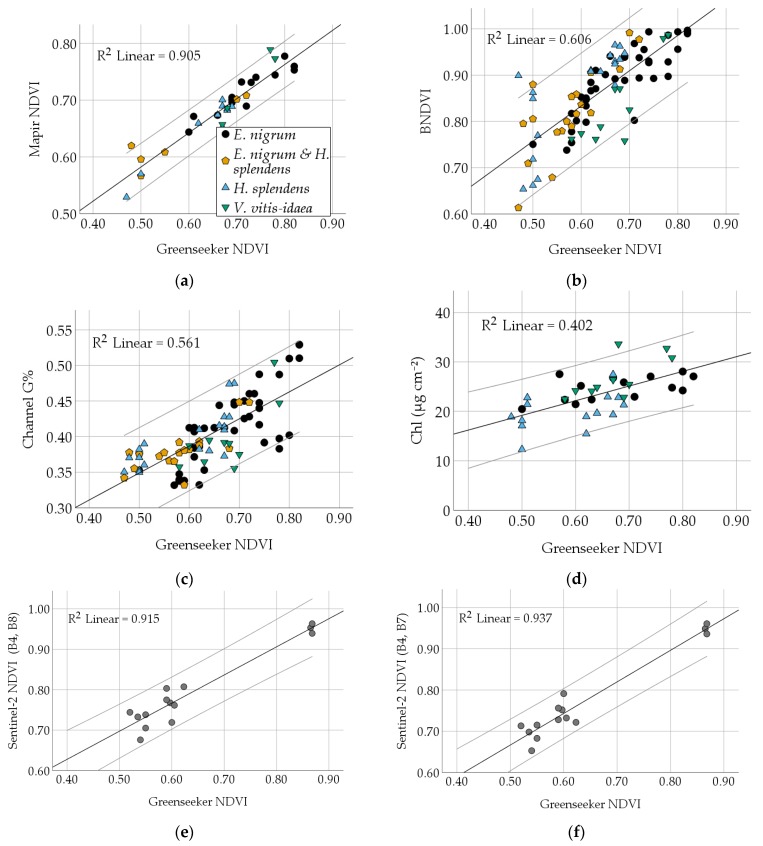
Species are marked with different symbols (see legend). 95% confidence intervals are grey. All correlations are significant (*p* < 0.001). The panels show the relationships between: (**a**) The active Greenseeker and the passive Mapir NDVI; (**b**) the BNDVI from the modified camera and NDVI; (**c**) Channel G% from the ordinary camera and NDVI; (**d**) chlorophyll content (Chl) and NDVI; (**e**) and (**f**) represent our satellite upscaling; comparing ground-based Greenseeker NDVI and Sentinel-2 NDVI, (note *n = 14*). NDVI is calculated with the named bands (= B). Equations and RMSE are shown in Table S4.

We assessed the use of the six greenness indices and the different calibration methods. Accuracy was not found to be improved by using a relatively cheap 3-step reflectance target instead of an ordinary gray card for white balance (Table S3). However, it was important to avoid overexposure of the calibration target. Comparing the greenness indices (Table 4): Mapir NDVI and Greenseeker NDVI were significantly correlated (*r* = 0.951, *p* < 0.001; Figure 1a), while ordinary RGB indices showed a much lower correlation, albeit still significant. NDVI to BNDVI (*r* = 0.779, *p* < 0.001) correlated slightly better than Channel G% to NDVI (*r* = 0.749, *p* < 0.001) and NDVI to GRVI (*r* = 0.689, *p* < 0.001). Greenseeker NDVI showed the best correlation with the chlorophyll content (*r* = 0.634; *p* < 0.001), while other indices, such as BNDVI, showed rather low correlations with the chlorophyll content (*r* = 0.433, *p* < 0.01; Table 4). Figure 1 illustrates the results for different species and how they correlate with the other indices. Comparison of BNDVI and Greenseeker NDVI (Figure 1b) reveals several high BNDVI values around 1 and increased deviation of lower values. Moreover, spaceborne NDVI data from the Sentinel-2 satellites based on band 4/7/8 (Figure 1e,f) are highly correlated with ground-sampled NDVI values (*r* = 0.956 and *r* = 0.968, *p* < 0.001, *n = 14*) obtained using the Greenseeker device. In principle, this allows for a significant upscaling from ground to space.

**Figure 2:**
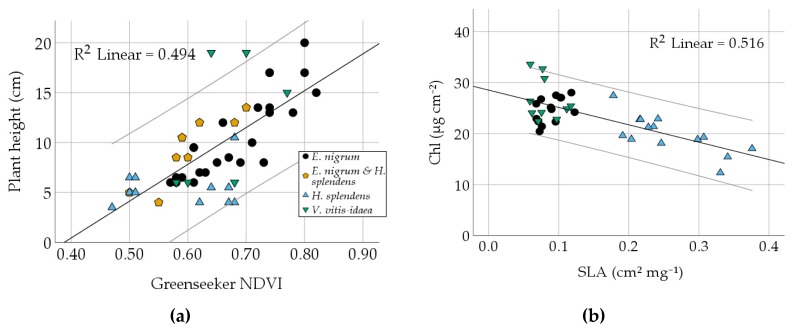
Relationship of two trait correlations from Table 4. Species are marked. 95% confidence intervals are grey. Relation between (**a**) median plant height and NDVI, (**b**) chlorophyll content and SLA. Equations and RMSE are shown in Table S4.

Joint analyses of all plant species resulted in variable correlations between the greenness indices and other plant traits, i.e. SLA, chlorophyll content, and plant height (Table 4). The nitrogen index (NBI) shows a good correlation with soil depth (*r* = 0.685, *p* < 0.001), indicating that the nutrients may be limited by shallow soil depths. The NDVI and the Channel G% indices allow for an assumption of plant height, as correlations are good (NDVI: *r* = 0.703, *p* < 0.001; Channel G%: *r* = 0.515, *p* < 0.001), even when comparing across functional groups, i.e. by considering mosses and dwarf shrubs together. Species-specific correlations are listed in Tables S5–S8. Specifically, the correlation (Figure 2b) between SLA and chlorophyll (*r* = −0.718, *p* < 0.001) is almost solely driven by the moss *H. splendens,* which shows a strong correlation when analyzed separately (*r* = −0.745, *p* < 0.01), whereas *V. vitis-idaea* and *E. nigrum* showed no significant correlation. A similar case is the SLA to flavonol correlation (*r* = −0.512, *p* = 0.001; Table 4). In this case, *E. nigrum* is the only species showing a significant correlation when analyzed species-wise (*r* = −0.589, *p* < 0.05), while the two other species showed no significant correlation.

### 3.3. Intraspecific Trait Variablility

**Figure 3:**
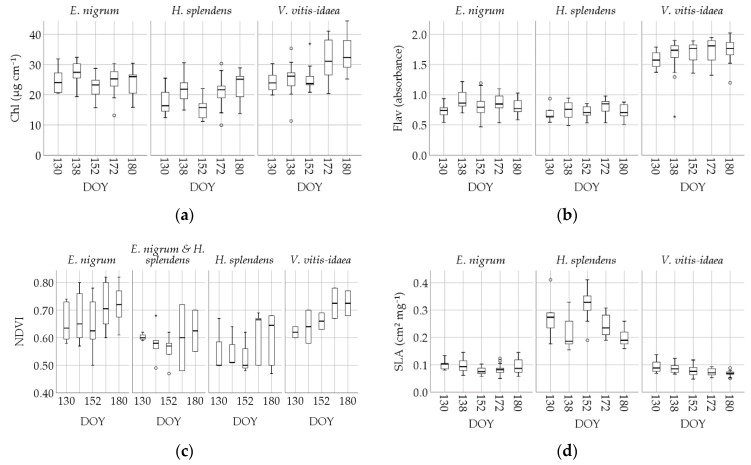
Intraspecific trait variability and its changes during the spring season for the species *Empterum nigrum*, *Vaccinium vitis-idaea,* and *Hylocomium splendens*. All species were monitored in varying health states, while each value represents one plot dominated by the named species. (**a**) Chlorophyll content (Chl); (**b**) flavonols (Flav); relative absorbance values; (**c**) Greenseeker NDVI; (**d**) SLA. These results were obtained at a moisture content (percent of wet weight) ranging from 55% to 75% for *H. splendens,* 63 to 68% for *V. vitis-idaea,* and 63 to 71% for *E. nigrum.*

Table 5 illustrates the overall trait variability during the study. Plant traits varied in the course of the study, with species showing contrasting responses (Figure 3). For example, Chl increased in *V. vitis-idaea,* but was stable in *E. nigrum* and *H. splendens* (Figure 3a), while the flavonol content was rather constant over time, but significantly higher in *V. vitis-idaea* than in the other two species (Figure 3b). In early June, however, all studied species showed a sudden decline in chlorophyll content (Figure 3a; DOY 152), (F_4,14_ = 7.945, *p* < 0.001), which coincided with the temperature dropping almost to freezing point and light snowfall during DOYs 150–151 (Figure S4). The post-hoc Bonferroni test confirms significant differences in chlorophyll content on the sampling dates before (DOY 138) and after (DOY 172) the temperature drop (*p* = 0.020 and *p* = 0.015). NDVI varied considerably within species (long boxes in Figure 3c) and only *V. vitis-idaea* showed a temporal trend in NDVI, which coincided with an increase in chlorophyll content (compare Figure 3a,c). SLA was constant over time, but significantly higher in the moss than in the two dwarf shrubs (Figure 3d). 

### 3.4. Suitable Traits for Stress Monitoring

**Figure 4:**
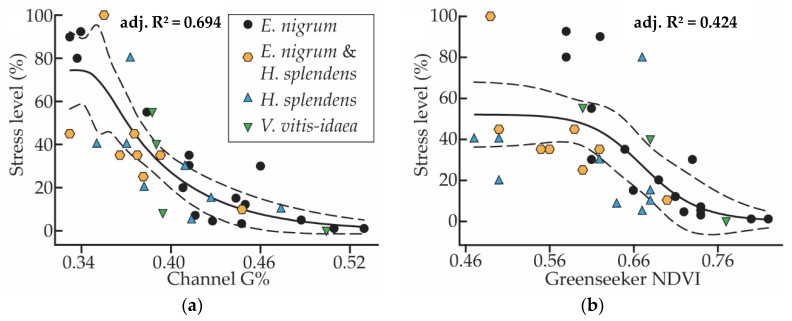
Comparison of stress level to greenness indices. 95% confidence intervals are dashed. Relation between (**a**) stress level and the RGB index Channel G%; (**b**) stress level and Greenseeker NDVI. Equations are shown in Table S4.

Our stress level estimate (Table 4) correlated with NDVI (*r* = −0.600, *p* < 0.001) and BNDVI (*r* = −0.654, *p* < 0.001), while the RGB indices performed best; *r* = 0.768, *p* < 0.001 for the Channel G% and *r* = −0.745, *p* < 0.001 for the GRVI, both as linear functions. Allowing a logistic relationship, a higher correlation level is obtained *r* = −0.833, *p* < 0.001 (Figure 4a) and r = −0.650, *p* < 0.001 (Figure 4b). Stress level estimates were also significantly correlated with plant height (*r* = −0.553, *p* = 0.001), as well as with soil depth (*r* = −0.336, *p* < 0.05). For litter, the relation with stress is reasonable, but not significant (*r* = −0.419, *p* = 0.084, *n* = 18). Neither flavonol absorbance nor chlorophyll content or the NBI readings could be related to the stress estimate (Table 4). No correlation was found for SLA and stress level (*n = 14*). 

## 4. Discussion

The range of intraspecific trait variability (1^st^ research question), is attributed well during the study. Some plant traits remained relatively stable during spring (SLA, Plant height, Flav), while others showed more variations during the season and to environmental circumstances (NDVI, ChannelG% and Chl). The small leaves of *E. nigrum* and shoots of *H. splendens* made the SLA measurements challenging. However, the infrequently used method that we decided to apply seemed to work well, as our results are comparable to SLA values retrieved in previous studies [48,49,50]. To our knowledge, our plots did not suffer from any major stress (browning) events during the last 3 years prior to our measurements, except that *Vaccinium myrtillus* in the area had been partly defoliated by larvae of geometrid moths [51], but this species was rare or absent in our plots. In the early growing season, plants are especially vulnerable to winter-related stress and are showing accumulated stress responses from the previous years [13,14]. Hence, we monitored the natural range of trait variabilities from start of the growing season (DOY 130) onwards. Chlorophyll content was dropping significantly when temperature fell to almost freezing point. However, more research is needed to validate this result. It might be that the slight snowfall, or both parameters jointly, instigated the decline in chlorophyll concentrations.

The second research question was to assess whether any of the studied plant traits are suitable for stress monitoring. Our data show that the stress level differed between plots; we found that plant height was related to soil depth and that soil depth was also related to NBI. Although we did not find any significant correlation between plant height and NBI, we assume that soil depth is a limiting factor for this ecosystem. Lower soil depth affects water and nutrient availability as well as soil temperature [52] and is also associated with areas of low snow accumulation during winter [13]. This is supported by the fact that the stress level decreased with increasing soil depth and that NDVI increased with increasing soil depth. 

In general, the flavonol content is associated with plant stress reactions [53]. However, we could not relate the flavonol absorbance to our stress level estimates. As the Dualex device estimates the flavonol content from spectral properties, it might not be able to measure the relevant flavonols in relation to the types of stress occurring in these subarctic plants. Dualex flavonol measurements are performed at the wavelengths 375 nm (UV-A) and 650 nm (red) [34]. This results in screening of mainly kaempferol, quercetin, and myricetin [53]. Our results are in agreement with Lefebvre et al. [54], who concluded that the Dualex device could not accurately predict the flavonol content in the three alpine plants they studied.

Concerning the third research question, our results show that ordinary RGB cameras may be used as NDVI surrogates and that they reflect various plant traits well. They performed equally well as modified cameras (with the infrared filter removed) for near-remote sensing approaches in the subarctic ecosystem. We found that a normal gray card, as used by professional photographers, was sufficient for the calibration process. Based on our findings, we recommend a simple white balance. Even if correlations to NDVI were slightly higher for BNDVI (*r* = 0.779) than for RGB greenness indices (0.689−0.749), one of the main strengths of the RGB cameras is that they are easier to operate than the modified devices. Sonnentag et al. [46] showed that different RGB cameras produce comparable results and that the choice of file format is not that important. Also, Nijland et al. [55] identified band separation and dynamic range as main problems when using converted cameras and therefore recommended the use of true color imaging. Another aspect is that the distribution of RGB cameras via smartphones is enormous and might be valuable for citizen science projects or app development [56]. In general, our greenness measurements are in agreement with existing reports on phenology at higher latitudes [30,57]. 

Moreover, the Channel G% index performed better than NDVI in characterizing some plant traits. This includes the stress level which showed a stronger correlation to Channel G% (linear: *r* = −0.768 vs. *r* = −0.600; logistic: *r* = −0.833 vs. *r* = −0.651) and NBI which showed a significant correlation (*r* = −0.354) to Channel G%, but not to NDVI. Consequently, the Channel G% index is of additional value for screening plant stress (2^nd^ research question). The significant correlation between RGB indices and chlorophyll meter readings (*r* = 0.38, *p* < 0.05) also implies that the RGB-based indices could be potential NDVI surrogates (see Table 4). In contrast to previous studies [28,29,58], our GNDVI data did not show any significant correlation with chlorophyll content or other plant traits. Correlations between chlorophyll and NDVI showed reasonable results [29], indicating that chlorophyll measurements are valid in spite of the untypical leaf structures of *H. splendens* and *E. nigrum*.

We found a high correlation between spaceborne NDVI and ground-sampled NDVI measured by the active Greenseeker device (maximum *r* = 0.968 for the Sentinel-2 NDVI calculated with bands 4 and 7). Nevertheless, despite of the strongly significant correlation, it is based only on 14 data points, implying that relationships have to be handled with care. It is a higher correlation than retrieved in previous studies, where near-remotely sensed NDVI data were compared to NDVI from Sentinel-2 and Landsat 8 [59,60]. A likely reason for the very strong correlation is that this study was carried out in a very open subarctic woodland (in parts nearly treeless and then considered as heath) where understory vegetation contributes very much to the NDVI detected by the satellites. We did not find major differences in the correlations, even when spectral properties (bandwidth and wavelength peaks) were not similar. This strongly suggests that active sensors can be used for validation of spaceborne data, for example, from Sentinel-2. 

## 5. Conclusions

The objective of this study was to assess the applicability of common plant traits and near-remote sensing approaches as tools to monitor the health state of dominant understory subarctic vegetation types that previously were shown to be vulnerable to winter climate change and other types of stress. In order to determine intraspecific trait variability, species were monitored in different health states. Due to this screening we are able to better validate the effect size of a browning event on the studied species. As the study was set in an area not recently damaged by stressful events, the different stress levels could be explained by differences in soil depth, which again act as a surrogate for several potential stressful elements, including moisture and nutrient deficits during the growing season and little snow protection during winter. Channel G% was the best RGB-based index in our study, and we recommend the use of this index. Finally, we found promising results by combining spaceborne Sentinel-2 data with the active near-remote sensor for measurements of NDVI. This could be a useful tool for upscaling the role of understory vegetation to the total NDVI measured by satellites in regions where browning occurs. Further research is recommended on the satellite upscaling, but also on the measured chlorophyll drop following a rapid midsummer temperature decrease to freezing point. Finally, we recommend following the same plots after a stressful weather event, to report direct as well as long-term changes in situ.

## Figures and Tables

**Table 2:** Comparison of spectral properties of the various NDVI devices listed in Table 1. Numbers in parentheses show the bandwidth in nanometers.

	Mapir	Greenseeker	Sentinel-2	Sentinel-2	Sentinel-2
	Survey 2 NDVI	Handheld Crop Sensor	Band 4	Band 7	Band 8
Red band	660 nm (50)	660 nm (25)	665 nm (30)		
NIR band	850 nm (70)	780 nm (25)		783 nm (20)	833 nm (106)
Spatial resolution	16 MP camera	an oval depending on the height of the sensor: at 60 cm, length is 25 cm	10 m	20 m	10 m

**Table 3:** Descriptive statistics of the monitored plant traits on plot level.

Name	Mean	Std. Error	Std. Deviation	N	5% PCTL	Median	95% PCTL	Skewness	Kurtosis
**Greenseeker NDVI**	0.64	0.0099	0.092	88	0.48	0.63	0.80	0.018	−0.701
**Mapir NDVI**	*0.69*	0.0104	0.062	35	0.56	0.69	0.78	−0.611	0.246
**BNDVI**	*0.86*	0.0096	0.090	88	0.68	0.87	0.99	−0.569	−0.233
**Channel G%**	*0.40*	0.0050	0.047	87	0.48	0.39	0.80	0.848	0.283
**GRVI**	*−0.098*	0.0087	0.081	88	−0.212	−0.108	0.072	0.659	0.349
**GNDVI**	*0.47*	0.0085	0.079	88	0.34	0.47	0.60	−0.395	0.393
**Plant height**	*9.46*	0.0638	4.508	50	4.00	8.25	19.00	0.748	−0.430
**Stress level**	*32.07*	4.6593	27.956	36	0.85	30.00	93.63	1.009	0.274
**Soil depth**	*13.73*	0.7238	6.790	88	4.00	12.00	25.00	0.267	−1.301
**SLA**	*0.149*	0.0151	0.093	38	0.059	0.103	0.343	0.993	−0.238
**Chl**	*23.46*	0.7151	4.401	38	15.28	22.92	32.78	−0.014	0.617
**Flav**	*1.01*	0.0672	0.414	38	0.61	0.81	1.86	1.065	−0.420
**NBI**	*25.31*	0.0151	7.241	38	14.34	25.93	36.04	−0.018	−1.166

**Table 4:** Correlations between greenness indices and plant traits at plot level. Root Mean Squared Error (RMSE) of the linear regressions (slope, intercept) is computed for significant correlations with *r* > 0.4. Chl = chlorophyll content, Flav = flavonol absorbance, and NBI = nitrogen balance index, i.e. the ratio between Chl and Flav.

		BNDVI	Channel G%	GRVI	GNDVI	Plant Height	Stress Level	Soil Depth	SLA	Chl	Flav	NBI
**NDVI**	Correlation	0.779	0.749	0.689	0.440	0.703	−0.600	0.454	−0.451	0.634	0.260	0.190
	Sig.	0.000	0.000	0.000	0.000	0.000	0.000	0.000	0.004	0.000	0.116	0.254
	RMSE	0.0571	0.0314	0.0592	0.0716	3.2404	22.682	6.086	0.0841	3.4572		
	Slope/ Intercept	0.762/0.376	0.379/0.159	0.606/−0.485	0.377/0.232	36.892/−14.313	−188.3/ 152.9	33.324/ −7.562	−0.446/ 0.436	29.706/ 4.320		
	N	88	87	88	88	50	36	88	38	38	38	38
**BNDVI**	Correlation		0.730	0.641	0.547	0.468	−0.654	0.246	−0.232	0.433	−0.029	0.327
	Sig.		0.000	0.000	0.000	0.001	0.000	0.021	0.161	0.007	0.863	0.045
	RMSE		0.0324	0.0627	0.0668	4.026	21.469			4.027		
	slope/intercept		0.378/0.076	0.576/ −0.595	0.480/0.059	26.478/ −13.720	−233.09/228.97			19.167/ 7.033		
	N		87	88	88	50	36	88	38	38	38	38
**Channel G%**	Correlation			0.909	0.454	0.515	−0.768	0.395	−0.141	0.376	−0.051	0.354
	Sig.			0.000	0.000	0.000	0.000	0.000	0.397	0.020	0.759	0.029
	RMSE			0.0340	0.0714	3.946	18.176					
	Slope/Intercept			1.565/ −0.728	0.164/0.768	49.185/ −10.268	−419.85/203.61					
	N			87	87	49	36	87	38	38	38	38
**GRVI**	Correlation				0.338	0.554	−0.745	0.393	−0.124	0.387	0.019	0.301
	Sig.				0.001	0.000	0.000	0.000	0.459	0.016	0.908	0.066
	RMSE					3.7909	18.909					
	slope/intercept					30.426/12.371	−246.65/11.770					
	N				88	50	36	88	38	38	38	38
**GNDVI**	Correlation					0.045	−0.220	0.070	−0.324	0.226	−0.120	0.294
	Sig.					0.758	0.196	0.515	0.047	0.172	0.472	0.074
	N					50	36	88	38	38	38	38
**Plant height**	Correlation						−0.553	0.425	−0.357	0.365	0.383	−0.160
	Sig.						0.001	0.002	0.103	0.095	0.079	0.477
	RMSE						22.129	6.3474				
	slope/intercept						−3.085/58.926	0.654/ 7.765				
	N						34	50	22	22	22	22
**Stress level**	Correlation							−0.336	−0.110	−0.232	−0.090	−0.021
	Sig.							0.045	0.707	0.425	0.760	0.944
	N							36	14	14	14	14
**Soil depth**	Correlation								−0.025	0.103	−0.441	0.685
	Sig.								0.883	0.539	0.006	0.000
	RMSE										0.3768	5.3469
	slope/intercept										−0.027/ 1.385	0.721/ 15.182
	N								38	38	38	38
**SLA**	Correlation									−0.718	−0.512	0.086
	Sig.									0.000	0.001	0.607
	RMSE									3.1102	0.3605	
	slope/intercept									−34.048/28.541	−2.282/ 1.353	
	N									38	38	38
**Chl**	Correlation										0.541	0.045
	Sig.										0.000	0.790
	RMSE										0.3530	
	slope/intercept										0.051/−0.180	
	N										38	38
**Flav**	Correlation											−0.787
	Sig.											0.000
	RMSE											4.5306
	slope/intercept											−13.759/39.239
	N											38

**Table 5:** Descriptive statistics of the monitored plant traits on species level.

Name	Specie	Mean	N	Std. Deviation	Std. Error	Median	5% PCTL	25% PCTL	75% PCTL	95% PCTL
Greenseeker NDVI	*E. nigrum*	0.65	59	0.094	0.012	0.62	0.49	0.58	0.72	0.82
	*H. splendens*	0.59	19	0.086	0.020	0.62	0.47	0.50	0.67	0.68 (90%)
	*V. vitis-idaea*	0.67	10	0.066	0.020	0.67	0.58	0.62	0.72	0.78 (90%)
Channel G%	*E. nigrum*	0.405	58	0.050	0.006	0.393	0.333	0.372	0.444	0.511
	*H. splendens*	0.396	19	0.037	0.008	0.383	0.350	0.383	0.415	0.474 (90%)
	*V. vitis-idaea*	0.397	10	0.046	0.015	0.389	0.355	0.363	0.408	0.497 (90%)
Plant height	*E. nigrum*	10.4	33	3.975	0.692	9.5	4,7	7.0	13.3	17.9
	*H. splendens*	5.5	11	1.955	0.589	5.0	3.5	4.0	6.5	9.7 (90%)
	*V. vitis-idaea*	11.8	6	6.55	2.676	10.5	6.0	6.0	19.0	-
SLA	*E. nigrum*	0.089	69	0.022	0.0027	0.085	0.057	0.072	0.105	0.130
	*H. splendens*	0.257	69	0.086	0.0104	0.246	0.164	0.189	0.291	0.411
	*V. vitis-idaea*	0.080	61	0.019	0.0024	0.079	0.052	0.066	0.092	0.117
Chl	*E. nigrum*	24.72	70	4.267	0.510	25.26	16.25	21.44	27.57	30.67
	*H. splendens*	19.88	69	5.122	0.617	20.01	11.90	15.59	23.88	28.54
	*V. vitis-idaea*	28.25	61	6.491	0.831	26.55	20.21	23.63	31.39	40.96
Flav	*E. nigrum*	0.833	69	0.164	0.020	0.818	0.542	0.727	0.943	1.145
	*H. splendens*	0.736	67	0.126	0.015	0.728	0.519	0.642	0.842	0.942
	*V. vitis-idaea*	1.671	61	0.232	0.030	1.706	1.297	1.558	1.843	1.944

## Supplementary Materials

The following are available online at https://www.mdpi.com/1424-8220/20/7/2102/s1, **Figure S1:** Study site of the 18 plots at the northern parts of Tromsøya (Norway). Exact coordinates are given in Table S1. **Figure S2:** Typical vegetation plots within the study area. (**a**) Detail view of one of the satellite upscaling areas. The photo was taken on the day of the upscaling experiment. Note: the snow patch on the left, which made plot selection difficult. The leafless trees show that this is from early spring prior to budbreak. (**b**) RGB image of a healthy plot (1 m^2^) of *E. nigrum*. (**c**) RGB image of a stressed plot of *E. nigrum*. **Figure S3:** Illustration of the working process for the different species. After measuring at least eight times with the chlorophyll meter (Chl, Flav, NBI), middle parts are hole-punched and cut and stored in sealed, numbered glasses to determine moisture content and SLA. **Figure S4:** Weather statistics for Tromsø from November 2016 to July 2017. The red line shows the mean value of daily temperature. The black line shows the average temperature from 1989-2018. Blue bars indicate the snow depth. Snow depth and long-time temperature data are from the Tromsø weather station (SN90450) located about 5.6 km south of the study area, while daily mean temperature is from the Stakkevollan weather station (SN90495) located about 900 m south of the main cluster of field plots. **Table S1:** Plot descriptions, including coordinates, stress level estimates, plant height, soil depth, litter, slope, and vegetation assessment. **Table S2:** Coordinates of the waypoints for satellite referencing. Waypoint coordinates are different from plot coordinates. **Table S3:** Plot-level relationships between different greenness indices and chlorophyll content for different calibration methods. Wb = white balance, 3-step = 3-step reflectance calibration. **Table S4:** Equations corresponding to the linear regressions in Figure 1 and Figure 2. **Table S5:** Correlation table for plots dominated by *E. nigrum*. Correlations are computed by Pearson’s correlation, while significance is two-tailed. **Table S6:** Correlation table for plots dominated by *Vaccinium vitis-idaea*. Correlations are computed by Pearson’s correlation, while significance is two-tailed. **Table S7:** Correlation table for plots dominated by *Hylocomium splendens*. Correlations are computed by Pearson’s correlation, while significance is two-tailed. **Table S8:** Correlation table for mixed plots of *Empetrum nigrum* and *Hylocomium splendens*. Correlations are computed by Pearson’s correlation, while significance is two-tailed.
