# Supplementary material for: Monitoring Winter Stress Vulnerability of High-Latitude Understory Vegetation Using Intraspecific Trait Variability and Remote Sensing Approaches
Source: Sensors (Basel). 2020 Apr 8;20(7):2102. doi: 10.3390/s20072102 (PMC7180702; doi:10.3390/s20072102)
Supplement: Supplementary file 1 [file sensors-20-02102-s001.pdf]

sensors

MDPI

Supplementary

Monitoring Winter Stress Vulnerability of High-Latitude Understory Vegetation Using Intraspecific Trait Variability and Remote Sensing Approaches

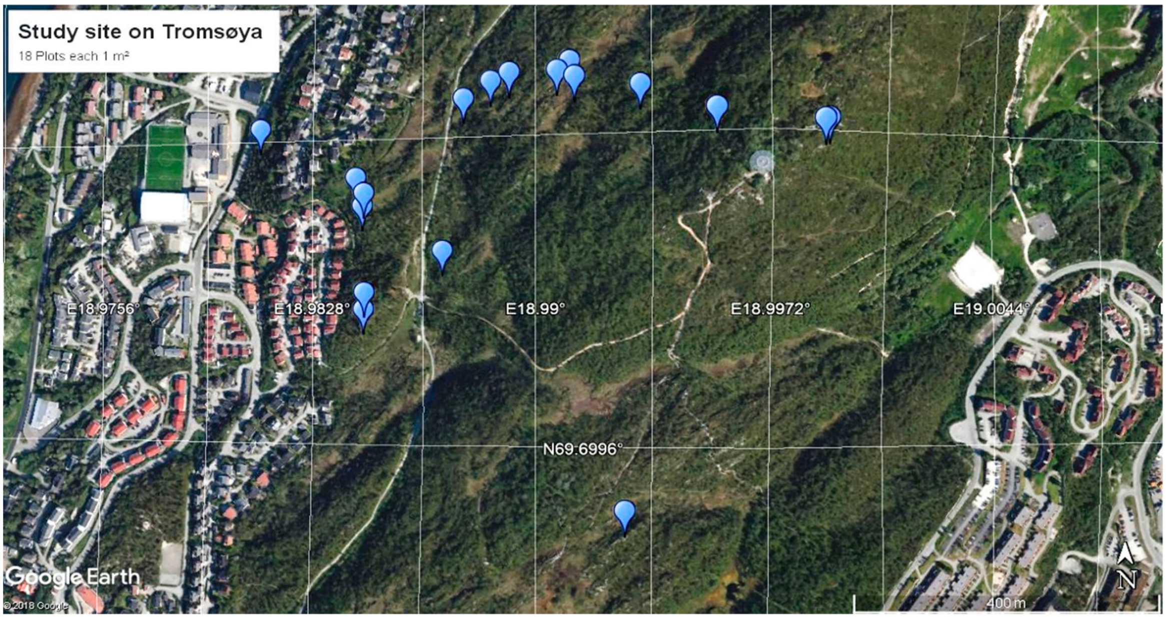

Figure S1. Study site of the 18 plots in the north of Tromsøya (Norway). Exact coordinates are given in Table S1.

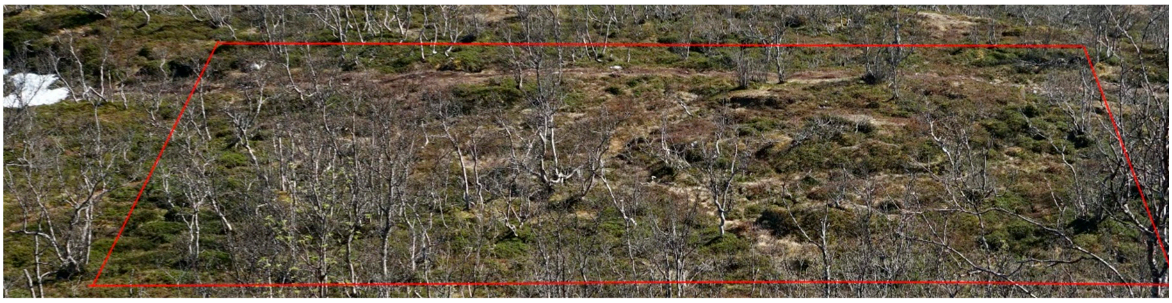

(**a**)

Figure S2. *Cont.*

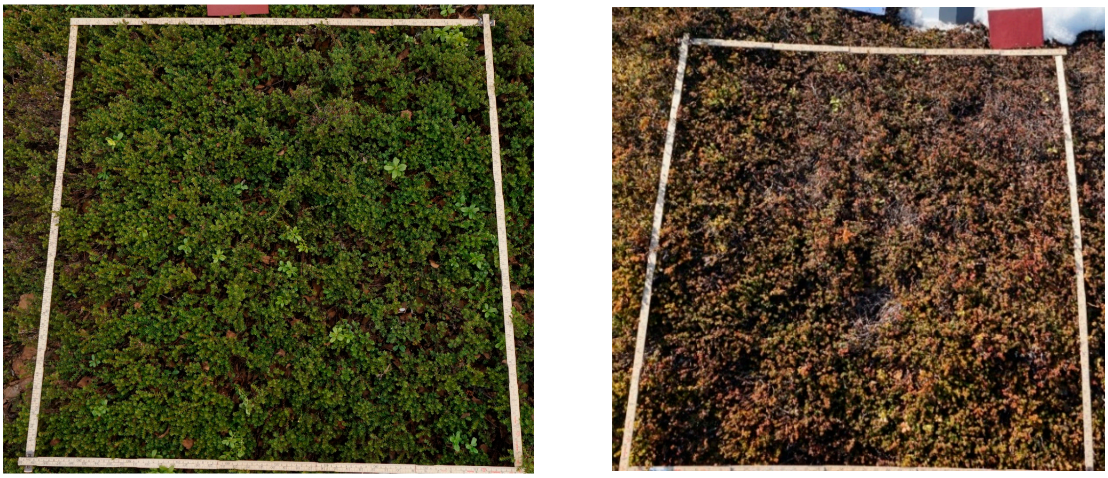

(**b**) (**c**)

Figure S2. Exemplary plots of the study. (**a**) shows one of satellite upscaling areas. The photo was taken on the day of the upscaling experiment. Note: the snow patch on the left, which made plot selection difficult. The leafless trees show that this is from early spring prior to budbreak. (**b**) RGB image of a healthy plot (1 m^2) of *E. nigrum*. (**c**) RGB image of a stressed plot of *E. nigrum*.

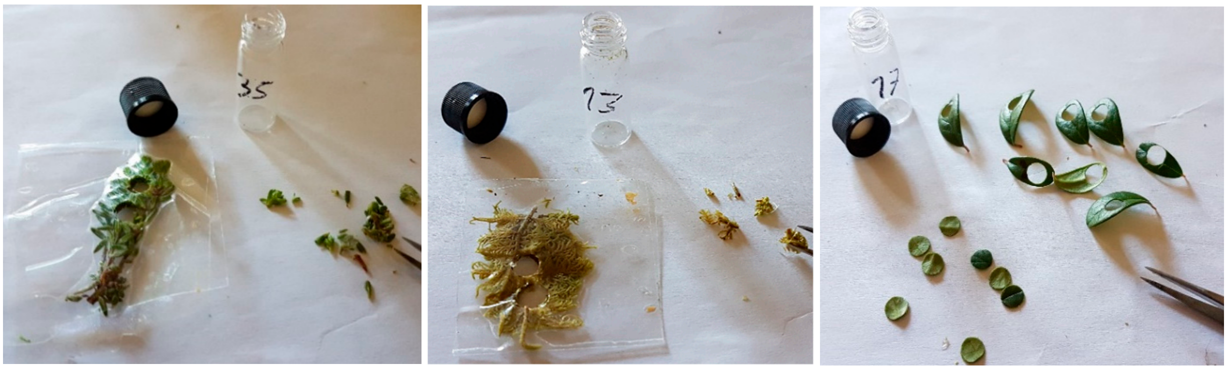

Figure S3. Illustration of the working process for the different species. After measuring at least eight times with the chlorophyll meter (Chl, Flav, NBI), middle parts are hole-punched and cut and stored in sealed, numbered glasses to determine moisture content and SLA.

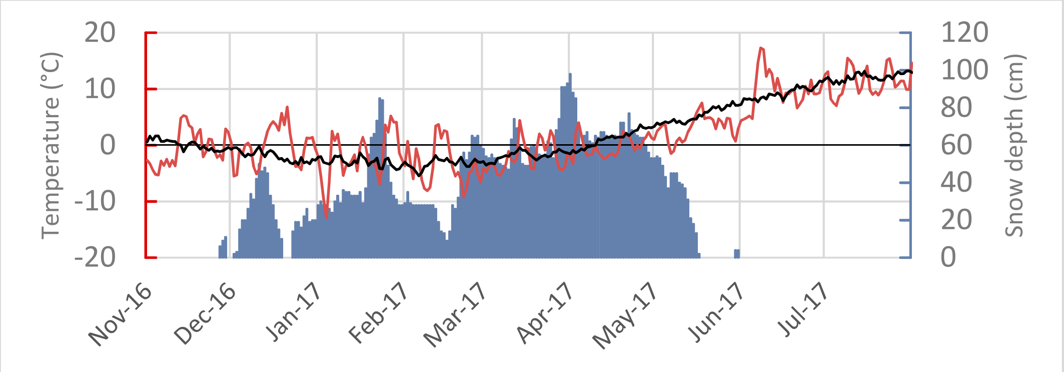

Figure S4. Weather statistics for Tromsø from November 2016 to July 2017. The red line shows the mean value of daily temperature. The black line shows the average temperature from 1989-2018. Blue bars indicate the snow depth. Snow depth and long-time temperature data are from the Tromsø weather station (SN90450) located about 5.6 km south of the study area, while daily mean temperature is from the Stakkevollan weather station (SN90495) located about 900 m south of the main cluster of field plots.

Table S1. Plot descriptions, including coordinates, stress estimate, plant height, soil depth, litter, slope, and vegetation assessment.

Plot No.	Dominant Species	GPS Coordinates (Lon/Lat)	Stress Estimate -Bare Eye- First week –Last week		Median height of Dominant Species [+ range] in cm	Soil depth Median [range]	%Litter	Cardinal Direction, Slope Inclination	Species Present in % (ground cover)
**1**	*Vaccinium vitis-idaea*	N 69.70306<br>E 018.98103°	8.5%	0%	19 [15–23]	12 [9–15 cm]	45%	SE, 15°	Ground layer (gl):<br>*H. splendens* <5 %<br>Dwarf layer (dw):<br>*V. myrtillus* 1 %*<br>*V. vitis-idaea* 60%<br>*Gymnocarpium dryopteris* <5 %**<br>*Cornus suecica* 10 %**
**2**	*Empetrum nigrum*	N 69.70245°<br>E 018.98426°	<5%	1%	17 [15–19]	>20	20%	SW, 25°	gl: not present<br>dw: *V. vitis-idaea* 5 %<br>*V. myrtillus* 1%*<br>*E. nigrum* 85 %
**3**	*Empetrum nigrum*	N 69.70227°<br>E 018.98452°	7%	1%	13 [10–15]	>20	17.5%	WSW, 22°	*Pleurozium schreberi* 1%<br>*H. splendens* 1 %<br>*V. vitis-idaea* 5 %<br>*V. myrtillus* 1%*<br>*E. nigrum* 85 %<br>*C. suecica* 5 %**
**4**	*Empetrum nigrum &*<br>*Hylocomium splendens*	N 69.70212°<br>E 018.98447°	35%	10%	12 [10–15]<br>& 4.5 [4–7]	20	20%	W, 28°	*P. schreberi* 45 %<br>*H. splendens* 20 %<br>*Peltigera* sp. 1 %<br>*V. vitis-idaea* <5 %<br>*V. myrtillus* 1%*<br>*E. nigrum* 40 %
**5**	*Hylocomium splendens*	N 69.70090°<br>E 018.98445°	20%	10%	5 [4–6]	20	7.5%	NNE, 2°	grass litter 5 %*<br>*H. splendens* 90 %<br>*V. vitis-idaea* <5 %<br>*V. myrtillus* < 1%*<br>*Pyrola minor* < 1 %<br>*C. suecica* 15 %**<br>*G. dryopteris* 5 %**
**6**	*Hylocomium splendens*	N 69.70110°<br>E 018.98449°	40%	5%	6.5 [4–10]	12 [8–20]	12. 5%	NNW, 13°	*H. splendens* 90 %<br>*V. vitis-idaea* <5 %<br>*V. myrtillus* 1%*<br>*E. nigrum* 5 %<br>*Lycopodium annotinum* <1 %<br>*C. suecica* 15 %**<br>*V. myrtillus* <5 %**
**7**	*Empetrum nigrum*	N 69.70159°<br>E 018.98702°	<5%	5%	13.5 [9–16]	20	10%	WSW, 21°	*P. schreberi* < 1 %<br>*H. splendens.* 5 %<br>*V. vitis-idaea* <5 %<br>*V. myrtillus* 1%*<br>*E. nigrum* 90 %

Table S1. *Cont.*

**8**	*Empetrum nigrum*	N 69.70336°<br>E 018.98772°	55%	15%	9.5 [7–11 cm]	10 [7–15 cm]	10%	SW, 21°	***P. schreberi* 10 %**<br>***H. splendens* 5 %**<br>***Arctous alpinae* < 1 %***<br>***Cladonia* sp. <1 %**<br>*V. vitis-idaea* <5 %<br>*V. myrtillus* 1%*<br>*E. nigrum* 90 %
**9**	*Empetrum nigrum*	N 69.70376°<br>E 018.99106°	80%	20%	6.5 [3–9]	10 [5–20]	15%	WNW, 15°	*P. schreberi* 5 %<br>*H. splendens* <5 %<br>*Dicranum spp.* <5 %<br>*Polytrichum* spp. 1 %<br>*Cladonia* spp. 5 %<br>*Rubus chamaemorus* <5 %**<br>*E. nigrum* 65 %
**10**	*Empetrum nigrum*	N 69.7058°<br>E 018.99121°	35%	12%	8.5 [7–10]	10 [5–20]	10%	SSW, 27°	*P. schreberi* 5 %<br>*H. splendens* 10 %<br>*Cladonia* spp. <5 %<br>*Andromeda polifolia* <1 %<br>*A. alpinae* < 5 %*<br>*V. myrtillus* <5 %*<br>*E. nigrum* 80 %
**11**	*Empetrum nigrum &*<br>*Hylocomium splendens*	N 69.70346°<br>E 018.99323°	25%	45%<br>(cut)	8.5 [5–14]<br>&<br>4 [3–6]	7.5 [5–15]	17.5%	WNW, 16°	*Dicranum* spp. 1 %<br>*H. splendens.* 70 %<br>*Linaceae* <5 %<br>*A. alpinae* 5 %*<br>*V. vitis-idaea* 15 %<br>*E. nigrum* 40 %
**12**	*Hylocomium splendens*	N 69.70316°<br>E 018.99550°	80%	30%	5.5 [3–7]	4.5 [3–6]	<5%	SW, 32°	*P. schreberi* 35 %<br>*H. splendens* 45 %<br>*Dicranum spp.* <5 %<br>*Polytrichum spp.* <5 %<br>*Cladonia spp.* <5 %<br>*V. vitis-idaea* 20 %<br>*Trientalis europaea* 25 %**
**13**	*Empetrum nigrum*	N 69.70303°<br>E 018.99885°	90 –<br>95%	30%	6 [4-8]	20	12.5%	SW, 6°	*P. schreberi* 20 %<br>*A. alpinae* 1 %*<br>*V. vitis-idaea* <5 %<br>*V. myrtillus* 1 %*<br>*E. nigrum* 90 %
**14**	*Empetrum nigrum*	N 69.70302°<br>E 018.99873°	90%	30%	7 [5-12]	20	10%	SSE, 23°	*Cladonia* spp. <1 %<br>*Dicranum* spp. <1 %<br>*P. schreberi* 1 %<br>*Calluna vulgaris* < 5 %<br>*V. vitis-idaea* <5 %<br>*V. myrtillus* 1 %*<br>*E. nigrum* 90 %

Table S1. *Cont.*

15	*Empetrum nigrum &*<br>*Hylocomium splendens*	N 69.70355°<br>E 018.98859°	45%	15%	12 [8–14 cm]<br>& 4.5 [4–6 cm]	4 [3–5 cm]	15%	W, 18°	***H. splendens*** **65 %**<br>***Dicranum*** **spp. <1 %**<br>***Peltigera*** **sp. 1 %**<br>***A. alpinae*** **1%***<br>*Vaccinium vitis-idaea* <5 %<br>*V. myrtillus* 1 %*<br>*E. nigrum* 45 %
**16**	*Vaccinium vitis-idaea*	N 69.69860°<br>E 018.99276°	55%	40%	6 [4–8]	6 [5–7]	25%	S, 10°	*A. alpinea* <1%*<br>*H. splendens* 15 %<br>*Racomitrium lanuginosum* 10 %<br>*V. vitis-idaea* 60 %<br>*V. myrtillus* 1 %*<br>*E. nigrum* <5 %<br>*V. myrtillus* 15 %**<br>*A. alpinea* 10%**
**17**	*Empetrum nigrum &*<br>*Hylocomium splendens*	N 69.70364°<br>E 018.98918°	35%	40%<br>(cut)	9 [7–11]<br>& 3 [2–5]	10 [6–15]	20%	W, 18°	*A. alpinae* 5%*<br>*H. splendens* 65 %<br>*Cladonia* spp. <1 %<br>*V. vitis-idaea* <5 %<br>*V. myrtillus** <1 %<br>*E. nigrum* 55 %
**18**	*Empetrum nigrum &*<br>*Hylocomium splendens*	N 69.70365°<br>E 018.99067°	100%	35%	5 [4–7]<br>& 3.5 [3–5]	9.5 [7–13]	10%	NNW, 30°	*A. alpinea* 1%*<br>*Andromeda polifolia* 1 %<br>*H. splendens* 60 %<br>*Peltigera* (lichen) 1 %<br>*Cladonia* spp. 10 %<br>*V. vitis-idaea* <5 %<br>*V. myrtillus* 5 %*<br>*E. nigrum* 40 %

* Deciduous plant; recognized on DOY 130 by the stem or last year's leaves.

** Deciduous plants greening up from DOY 172.

Table S2. Coordinates of the waypoints for satellite referencing. Waypoint coordinates are different from plot coordinates.

Latitude °N	Longitude °E
69.70155	18.99287
69.70138	18.99220
69.70113	18.99212
69.70111	18.99301
69.70139	18.99338
69.70193	18.99586
69.70164	18.99623
69.70170	18.99757
69.70212	18.99696
69.65089	18.90440
69.65097	18.90495
69.65072	18.90494
69.65070	18.90422
69.65083	18.90459

Table S3. Plot-level relationships between different greenness indices and chlorophyll content for different calibration methods. Wb = white balance, 3-step = 3-step reflectance calibration.

		Mapir NDVI	Channel G% (wb)	Channel G% (3-step)	BNDVI (wb)	GRVI (3-step)	GNDVI (wb)	Chlorophyll Content
**Greenseeker NDVI**	Correlation	0.951	0.749	0.725	0.779	0.689	0.440	0.634
	Sig.	0.000	0.000	0.000	0.000	0.000	0.000	0.000
	N	35	87	87	88	88	88	38
**Mapir NDVI**	Correlation		0.880	0.889	0.775	0.859	−0.002	0.557
	Sig.		0.000	0.000	0.000	0.000	0.993	0.025
	N		35	35	35	35	35	16
**Channel G%** (white balance)	Correlation			0.972	0.730	0.909	0.454	0.376
	Sig.			0.000	0.000	0.000	0.000	0.020
	N			87	87	87	87	38
**Channel G%** (3-step)	Correlation				0.746	0.954	0.477	0.374
	Sig.				0.000	0.000	0.000	0.021
	N				87	87	87	38
**BNDVI** (white balance)	Correlation					0.641	0.547	0.433
	Sig.					0.000	0.000	0.007
	N					88	88	38
**GRVI** (3-step)	Correlation						0.338	0.387
	Sig.						0.001	0.016
	N						88	38
**GNDVI** (white balance)	Correlation							0.226
	Sig.							0.172
	N							38

Table S4. Equations corresponding to the linear regressions in Figures 1, 2 and 4.

Figure, Panel	Equation
1, a	y = 0.28 + 0.60x<br>RMSE = 0.01937
1, b	y = 0.38 + 0.76x<br>RMSE = 0.0571
1, c	y = 0.16 + 0.38x<br>RMSE = 0.0314
1, d	y = 4.32 + 29.71x<br>RMSE = 3.4572
1, e	y = 0.350 + 0.696x,<br>RMSE = 0.02850
1, f	y = 0.284 + 0.766x,<br>RMSE = 0.02669
2, a	y = −14.31 + 36.89x<br>RMSE = 3.2404
2, b	y = 28.54 - 34.05x<br>RMSE = 3.1102
4, a	y= 74.66/(1 + exp(−80.79(−229.73 × x))^(1/11.01))
4, b	y = 1/(0.019 + (62.051 × (x^20.470)))

Table S5. Correlation table for plots dominated by *E. nigrum*. Correlations are computed by Pearson's correlation, while significance is two-tailed.

		Greenseeker NDVI	BNDVI	Channel G%	GRVI	GNDVI	Mapir NDVI	Plant Height	Stress Level	Soil Depth	SLA	NBI	Flav	Litter	Moisture	Chl
**Greenseeker NDVI**	Correlation	1	0.849**	0.774**	0.719**	0.489**	0.936**	0.851**	−0.837**	0.575**	0.554*	0.637*	−0.282	0.520*	0.512	0.558*
	Sig.		0.000	0.000	0.000	0.001	0.000	0.000	0.000	0.000	0.040	0.014	0.328	0.039	0.061	0.038
	N	40	40	39	40	40	15	24	16	40	14	14	14	16	14	14
**BNDVI**	Correlation		1	0.831**	0.772**	0.569**	0.892**	0.782**	−0.841**	0.304	0.256	0.297	−0.066	0.218	0.410	0.328
	Sig.			0.000	0.000	0.000	0.000	0.000	0.000	0.057	0.377	0.303	0.822	0.417	0.146	0.252
	N		40	39	40	40	15	24	16	40	14	14	14	16	14	14
**Channel G%**	Correlation			1	0.918**	0.700**	0.875**	0.726**	−0.873**	0.292	0.252	0.216	−0.040	0.156	0.545*	0.232
	Sig.				0.000	0.000	0.000	0.000	0.000	0.071	0.385	0.458	0.891	0.578	0.044	0.424
	N			39	39	39	15	23	16	39	14	14	14	15	14	14
**GRVI**	Correlation				1	0.618**	0.827**	0.750**	−0.868**	0.355*	0.414	0.364	−0.192	0.326	0.612*	0.261
	Sig.					0.000	0.000	0.000	0.000	0.025	0.141	0.201	0.511	0.218	0.020	0.368
	N				40	40	15	24	16	40	14	14	14	16	14	14
**GNDVI**	Correlation					1	0.110	0.181	−0.475	0.030	−0.078	0.088	−0.032	0.060	0.233	0.052
	Sig.						0.696	0.398	0.063	0.854	0.791	0.764	0.913	0.825	0.422	0.859
	N					40	15	24	16	40	14	14	14	16	14	14
**Mapir NDVI**	Correlation						1	0.745*	−0.692	0.512	0.643	0.588	−0.292	c	0.671	0.455
	Sig.							0.034	0.057	0.051	0.168	0.219	0.575		0.145	0.364
	N						15	8	8	15	6	6	6	0	6	6
**Plant height**	Correlation							1	−0.744**	0.549**	0.691	0.469	−0.256	0.569*	0.685	0.226
	Sig.								0.001	0.005	0.058	0.242	0.541	0.021	0.061	0.591
	N							24	16	24	8	8	8	16	8	8
**Stress level**	Correlation								1	−0.297	−0.160	0.296	−0.670	−0.403	−0.483	−0.597
	Sig.									0.263	0.797	0.629	0.216	0.322	0.410	0.288
	N								16	16	5	5	5	8	5	5

Table S5. *Cont.*

**Soil depth**	Correlation								1	0.811**	0.737**	−0.669**	0.552*	0.521	0.234
	Sig.									0.000	0.003	0.009	0.027	0.056	0.421
	N								40	14	14	14	16	14	14
**SLA**	Correlation									1	0.816**	−0.589*	0.859	0.811**	0.493
	Sig.										0.000	0.027	0.062	0.000	0.074
	N									14	14	14	5	14	14
**NBI**	Correlation										1	−0.789**	0.738	0.574*	0.466
	Sig.											0.001	0.155	0.032	0.093
	N										14	14	5	14	14
**Flav**	Correlation											1	−0.393	−0.361	0.157
	Sig.												0.513	0.205	0.592
	N											14	5	14	14
**Litter**	Correlation												1	0.976**	0.396
	Sig.													0.004	0.510
	N												16	5	5
**Moisture**	Correlation													1	0.432
	Sig.														0.123
	N													14	14
**Chl**	Correlation														1
	Sig.														
	N														14

** Correlation is significant at the 0.01 level (2-tailed). * Correlation is significant at the 0.05 level (2-tailed). c Cannot be computed, because at least one of the variables is constant.

Table S6. Correlation table for plots dominated by *Vaccinium vitis-idaea*. Correlations are computed by Pearson's correlation, while significance is two-tailed.

		**Greenseeker NDVI**	**BNDVI**	**Channel G%**	**GRVI**	**GNDVI**	**Mapir NDVI**	**Plant Height**	**Stress Level**	**Soil Depth**	**SLA**	**NBI**	**Flav**	**Litter**	**Moisture**	**Chl**
**Greenseeker NDVI**	Correlation	1	0.865**	0.746*	0.721*	−0.340	0.979*	0.520	−0.700	0.674*	0.146	0.121	0.902**	0.873	−0.285	0.709*
	Sig.		0.001	0.013	0.019	0.336	0.021	0.291	0.300	0.033	0.688	0.739	0.000	0.127	0.425	0.022
	N	10	10	10	10	10	4	6	4	10	10	10	10	4	10	10
**BNDVI**	Correlation		1	0.885**	0.850**	−0.316	0.973*	0.234	−0.573	0.361	−0.171	0.446	0.648*	0.820	−0.117	0.858**
	Sig.			0.001	0.002	0.373	0.027	0.655	0.427	0.305	0.636	0.197	0.043	0.180	0.747	0.001
	N		10	10	10	10	4	6	4	10	10	10	10	4	10	10
**Channel G%**	Correlation			1	0.938**	−0.663*	0.923	0.289	−0.705	0.424	−0.080	0.339	0.585	0.446	−0.064	0.751*
	Sig.				0.000	0.037	0.077	0.579	0.295	0.222	0.826	0.338	0.075	0.554	0.861	0.012
	N			10	10	10	4	6	4	10	10	10	10	4	10	10
**GRVI**	Correlation				1	−0.609	0.981*	0.548	−0.862	0.543	0.149	0.261	0.606	0.708	0.172	0.690*
	Sig.					0.062	0.019	0.260	0.138	0.104	0.682	0.467	0.063	0.292	0.634	0.027
	N				10	10	4	6	4	10	10	10	10	4	10	10
**GNDVI**	Correlation					1	−0.901	−0.411	0.819	−0.571	−0.276	0.140	−0.305	−0.129	0.128	−0.142
	Sig.						0.099	0.418	0.181	0.085	0.440	0.699	0.391	0.871	0.724	0.696
	N					10	4	6	4	10	10	10	10	4	10	10
**Mapir NDVI**	Correlation						1	1.000**	−1.000**	0.978*	0.965*	−0.235	0.931	.c	−0.927	0.503
	Sig.									0.022	0.035	0.765	0.069		0.073	0.497
	N						4	2	2	4	4	4	4	0	4	4
**Plant height**	Correlation							1	−0.899	0.975**	0.888*	−0.475	0.639	1.000**	0.096	−0.026
	Sig.								0.101	0.001	0.018	0.341	0.172	0.000	0.856	0.962
	N							6	4	6	6	6	6	4	6	6
**Stress level**	Correlation								1	−0.962*	−0.522	0.083	−0.671	−1.000**	0.605	−0.255
	Sig.									0.038	0.478	0.917	0.329		0.395	0.745
	N								4	4	4	4	4	2	4	4
**Soil depth**	Correlation									1	0.783**	−0.402	0.780**	1.000**	−0.008	0.152
	Sig.										0.007	0.250	0.008	0.000	0.983	0.674
	N									10	10	10	10	4	10	10

Table S6. *Cont.*

**SLA**	Correlation										1	−0.589	0.401	0.992**	0.395	−0.294
	Sig.											0.073	0.251	0.008	0.258	0.410
	N										10	10	10	4	10	10
**NBI**	Correlation											1	−0.226	−0.567	−0.079	0.763*
	Sig.												0.530	0.433	0.828	0.010
	N											10	10	4	10	10
**Flav**	Correlation												1	0.711	−0.084	0.450
	Sig.													0.289	0.817	0.192
	N												10	4	10	10
**Litter**	Correlation													1	0.075	0.819
	Sig.														0.925	0.181
	N													4	4	4
**Moisture**	Correlation														1	−0.135
	Sig.															0.711
	N														10	10
**Chl**	Correlation															1
	Sig.															
	N															10

**. Correlation is significant at the 0.01 level (2-tailed). *. Correlation is significant at the 0.05 level (2-tailed). c Cannot be computed, because at least one of the variables is constant.

Table S7. Correlation table for plots dominated by *Hylocomium splendens*. Correlations are computed by Pearson's correlation, while significance is two-tailed.

		Greenseeker NDVI	BNDVI	Channel G%	GRVI	GNDVI	Mapir NDVI	Plant Height	Stress Level	Soil Depth	SLA	NBI	Flav	Litter	Moisture	Chl
**Greenseeker NDVI**	Correlation	1	0.745**	0.775**	0.611**	0.587**	0.966**	0.191	−0.215	−0.292	−0.424	0.384	0.090	−0.843*	−0.377	0.465
	Sig.		0.000	0.000	0.003	0.005	0.000	0.553	0.579	0.199	0.130	0.175	0.758	0.035	0.184	0.094
	N	21	21	21	21	21	12	12	9	21	14	14	14	6	14	14
**BNDVI**	Correlation		1	0.550**	0.397	0.775**	0.797**	0.077	−0.232	−0.490*	−0.474	0.335	0.142	−0.375	−0.438	0.465
	Sig.			0.010	0.075	0.000	0.002	0.812	0.549	0.024	0.087	0.241	0.628	0.464	0.117	0.094
	N		21	21	21	21	12	12	9	21	14	14	14	6	14	14
**Channel G%**	Correlation			1	0.881**	0.500*	0.860**	0.056	−0.652	0.200	−0.377	0.710**	−0.299	−0.493	−0.535*	0.500
	Sig.				0.000	0.021	0.000	0.862	0.057	0.384	0.183	0.004	0.300	0.320	0.049	0.069
	N			21	21	21	12	12	9	21	14	14	14	6	14	14
**GRVI**	Correlation				1	0.213	0.749**	−0.095	−0.434	0.301	−0.153	0.645*	−0.469	−0.201	−0.456	0.350
	Sig.					0.355	0.005	0.770	0.243	0.184	0.602	0.013	0.091	0.703	0.102	0.220
	N				21	21	12	12	9	21	14	14	14	6	14	14
**GNDVI**	Correlation					1	0.670*	0.143	−0.362	−0.457*	−0.483	0.268	0.426	0.013	−0.348	0.531
	Sig.						0.017	0.658	0.339	0.037	0.080	0.354	0.129	0.981	0.223	0.051
	N					21	12	12	9	21	14	14	14	6	14	14
**Mapir NDVI**	Correlation						1	0.308	−0.863*	0.189	−0.696	0.815*	−0.218	.c	−0.647	0.860*
	Sig.							0.553	0.027	0.556	0.124	0.048	0.678		0.165	0.028
	N						12	6	6	12	6	6	6	0	6	6
**Plant height**	Correlation							1	−0.044	−0.337	0.162	−0.575	0.282	0.677	0.223	−0.349
	Sig.								0.910	0.284	0.702	0.136	0.498	0.139	0.596	0.396
	N							12	9	12	8	8	8	6	8	8
**Stress level**	Correlation								1	−0.489	0.204	−0.803	−0.069	−0.631	0.078	−0.788
	Sig.									0.182	0.742	0.102	0.913	0.565	0.901	0.114
	N								9	9	5	5	5	3	5	5
**Soil depth**	Correlation									1	0.084	0.512	−0.437	0.457	−0.131	0.204
	Sig.										0.776	0.061	0.118	0.362	0.656	0.485
	N									21	14	14	14	6	14	14

Table S7. *Cont.*

**SLA**	Correlation										1	−0.663**	−0.208	0.050	0.850**	−0.745**
	Sig.											0.010	0.476	0.937	0.000	0.002
	N										14	14	14	5	14	14
**NBI**	Correlation											1	−0.343	−0.015	−0.786**	0.795**
	Sig.												0.230	0.981	0.001	0.001
	N											14	14	5	14	14
**Flav**	Correlation												1	0.395	0.110	0.253
	Sig.													0.511	0.708	0.384
	N												14	5	14	14
**Litter**	Correlation													1	−0.020	0.195
	Sig.														0.974	0.753
	N													6	5	5
**Moisture**	Correlation														1	−0.669**
	Sig.															0.009
	N														14	14
**Chl**	Correlation															1
	Sig.															
	N															14

**. Correlation is significant at the 0.01 level (2-tailed). *. Correlation is significant at the 0.05 level (2-tailed). c. Cannot be computed, because at least one of the variables is constant.

Table S8. Correlation table for mixed plots of *Empetrum nigrum* and *Hylocomium splendens*. Correlations are computed by Pearson's correlation, while significance is two-tailed.

		Greenseeker NDVI	BNDVI	Channel G%	GRVI	GNDVI	Mapir NDVI	Plant Height	Stress Level	Soil Depth	Litter
**Greenseeker NDVI**	Correlation	1	0.892**	0.755**	0.640**	0.532*	0.944	0.826*	−0.832*	0.621**	0.744*
	Sig.		0.000	0.000	0.006	0.028	0.056	0.011	0.020	0.008	0.034
	N	17	17	17	17	17	4	8	7	17	8
**BNDVI**	Correlation		1	0.727**	0.643**	0.592*	0.995**	0.859**	−0.751	0.542*	0.713*
	Sig.			0.001	0.005	0.012	0.005	0.006	0.052	0.025	0.047
	N		17	17	17	17	4	8	7	17	8
**Channel G%**	Correlation			1	0.837**	0.667**	0.995**	0.407	−0.610	0.633**	0.558
	Sig.				0.000	0.003	0.005	0.317	0.145	0.006	0.151
	N			17	17	17	4	8	7	17	8
**GRVI**	Correlation				1	0.740**	0.996**	0.379	−0.544	0.492*	0.082
	Sig.					0.001	0.004	0.355	0.207	0.045	0.847
	N				17	17	4	8	7	17	8
**GNDVI**	Correlation					1	0.540	−0.183	−0.438	0.347	0.232
	Sig.						0.460	0.665	0.326	0.173	0.581
	N					17	4	8	7	17	8
**Mapir NDVI**	Correlation						1	1.000**	−1.000**	0.995**	c
	Sig.									0.005	
	N						4	2	2	4	0
**Plant Height**	Correlation							1	−0.385	0.537	0.427
	Sig.								0.523	0.170	0.398
	N							8	5	8	6
**Stress Level**	Correlation								1	−0.361	−0.894*
	Sig.									0.427	0.041
	N								7	7	5
**Soil Depth**	Correlation									1	0.551
	Sig.										0.157
	N									17	8

Table S8. *Cont.*

**Litter**	Correlation										1
	Sig.										
	N										8

**. Correlation is significant at the 0.01 level (2-tailed). *. Correlation is significant at the 0.05 level (2-tailed). c Cannot be computed, because at least one of the variables is constan
